# Feasibility of deep learning algorithm in diagnosing lumbar central canal stenosis using abdominal CT

**DOI:** 10.1007/s00256-024-04796-z

**Published:** 2024-09-09

**Authors:** Yejin Jeon, Bo Ram Kim, Hyoung In Choi, Eugene Lee, Da-Wit Kim, Boorym Choi, Joon Woo Lee

**Affiliations:** 1 Department of Radiology, Seoul National University Bundang Hospital, 82 Gumi-ro, 173 Beon-Gil, Bundang-Gu, Seongnam-Si, Gyeonggi-Do 13620 Republic of Korea; 2 Coreline Soft Co. Ltd., World-Cup Bukro 6-Gil, Mapogu, Seoul, 03991 Korea; 3 Department of Radiology, College of Medicine, Seoul National University, 103, Daehak-Ro, Jongno-Gu, Seoul, 03080 Republic of Korea

**Keywords:** Computed tomography, Lumbar spine, Central canal stenosis, Artificial Intelligence, Deep learning

## Abstract

**Objective:**

To develop a deep learning algorithm for diagnosing lumbar central canal stenosis (LCCS) using abdominal CT (ACT) and lumbar spine CT (LCT).

**Materials and methods:**

This retrospective study involved 109 patients undergoing LCTs and ACTs between January 2014 and July 2021. The dural sac on CT images was manually segmented and classified as normal or stenosed (dural sac cross-sectional area ≥ 100 mm^2^ or < 100 mm^2^, respectively). A deep learning model based on U-Net architecture was developed to automatically segment the dural sac and classify the central canal stenosis. The classification performance of the model was compared on a testing set (990 images from 9 patients). The accuracy, sensitivity, and specificity of automatic segmentation were quantitatively evaluated by comparing its Dice similarity coefficient (DSC) and intraclass correlation coefficient (ICC) with those of manual segmentation.

**Results:**

In total, 990 CT images from nine patients (mean age ± standard deviation, 77 ± 7 years; six men) were evaluated. The algorithm achieved high segmentation performance with a DSC of 0.85 ± 0.10 and ICC of 0.82 (95% confidence interval [CI]: 0.80,0.85). The ICC between ACTs and LCTs on the deep learning algorithm was 0.89 (95%CI: 0.87,0.91). The accuracy of the algorithm in diagnosing LCCS with dichotomous classification was 84%(95%CI: 0.82,0.86). In dataset analysis, the accuracy of ACTs and LCTs was 85%(95%CI: 0.82,0.88) and 83%(95%CI: 0.79,0.86), respectively. The model showed better accuracy for ACT than LCT.

**Conclusion:**

The deep learning algorithm automatically diagnosed LCCS on LCTs and ACTs. ACT had a diagnostic performance for LCCS comparable to that of LCT.

## Introduction

Lumbar spinal stenosis is a major cause of lower back pain. Over the last few decades, diagnosis and surgery rates for lumbar spinal stenosis have increased substantially because of improved diagnostic methods and increased life expectancy [1]. The high incidence of lumbar spinal stenosis has resulted in substantial societal costs from associated treatment and disease [2]. Abdominal CT is a frequently used imaging modality for various clinical indications, and not surprisingly, many CT scans are performed in patients who also have low back pain. Although abdominal CT is not performed to evaluate the lumbar spine, patients with lumbar spinal stenosis, especially older patients with vague symptoms, often undergo an abdominal CT. However, radiologic findings of lumbar spinal stenosis are often missed on abdominal CT scans. MRI is the imaging modality for evaluating spinal disorders because of its excellent soft tissue contrast; however, with technological advancement, multidetector CT (MDCT) with post-processed thin section sagittal reformations has allowed the evaluation of the lumbar spine on abdominal CT studies. If lumbar spinal stenosis can be assessed on abdominal CT, patients with lumbar spinal stenosis could receive an explanation for their symptoms without the need for additional costly evaluations.

Many studies have been conducted to diagnose and classify lumbar spinal stenosis. Schonstrom defined no spinal stenosis as dural sac cross-sectional area (DSA) of > 100 mm^2^, relative lumbar spinal stenosis as DSA of 75–99 mm^2^, and absolute lumbar spinal stenosis as DSA of 0–74 mm^2^ [3–5]. In a systematic review, most studies reported central canal stenosis when DSA was < 100 mm^2^ [4–9]. Therefore, in this study, the data were divided into two groups: normal (DSA ≥ 100 mm^2^) and stenosis (DSA < 100 mm^2^).

Meanwhile, deep learning technology plays an important role in the analysis of medical images [10–15]. Recent studies have shown that deep learning models can detect and classify lumbar spinal stenosis [16–18]. However, most deep learning-based studies have focused on MRI. Therefore, in this study, we aimed to develop a deep learning algorithm for diagnosing central canal stenosis of the lumbar spine using both lumbar and abdominal CTs and compare the diagnostic performance of lumbar and abdominal CTs.

## Materials and methods

This study was approved by the Institutional Review Board of our hospital (B-2109–708-101). The requirement for informed consent was waived by the review board.

### Dataset and labeling

**Fig. 1:**
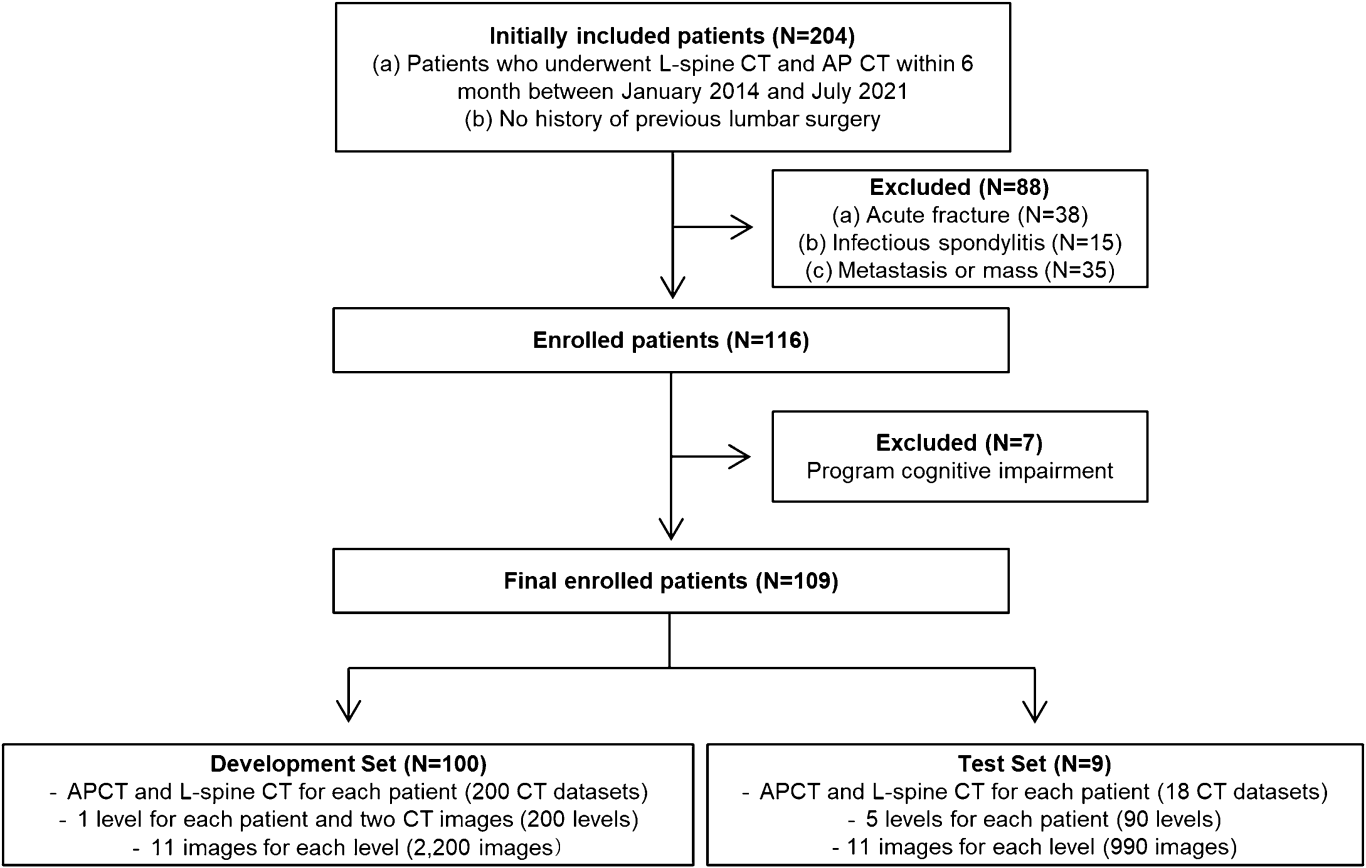
Flowchart of datasets for development and test. L-spine CT = Lumbar spine CT, AP CT = Abdominal CT

**Fig. 2:**
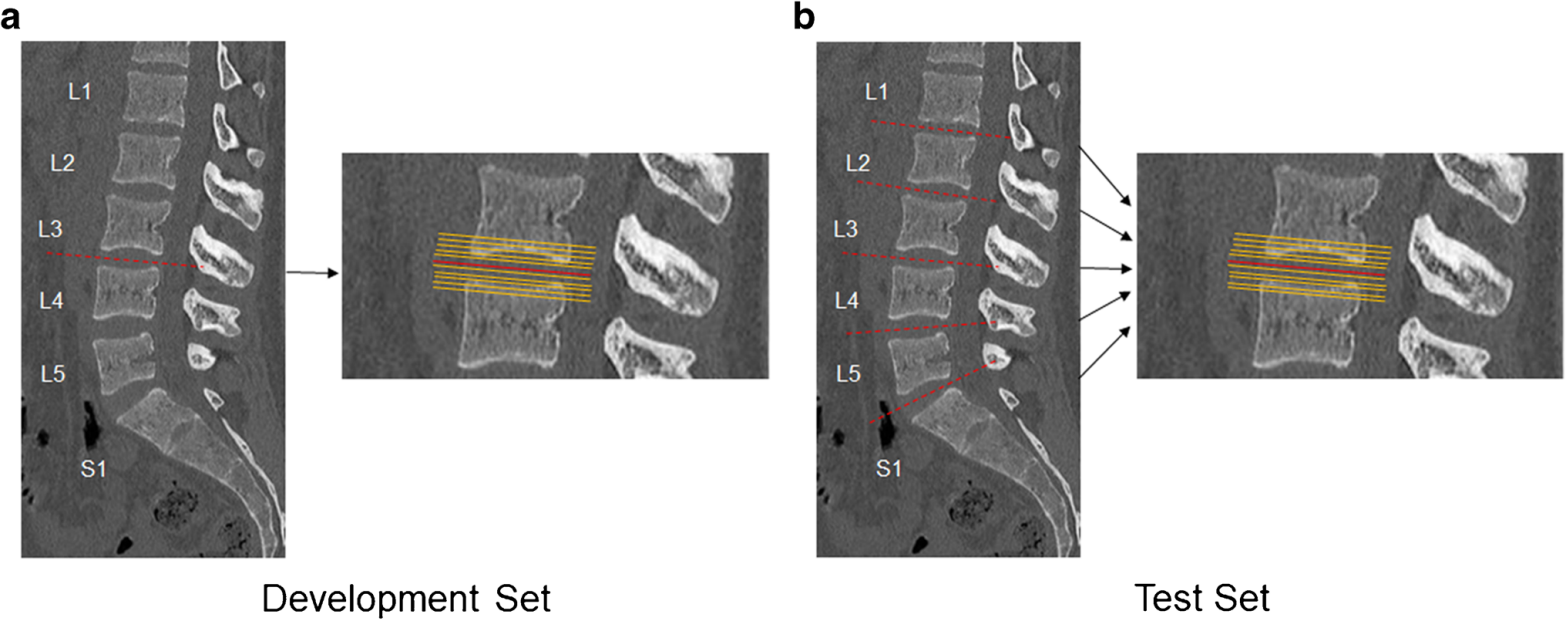
Development and test datasets. In the development set (**a**), one disc level for each patient was randomly assigned to L1-L2, L2-L3, L3-L4, L4-L5, or L5-S1. In the test set (**b**), all disc levels for each patient were included. For each level, a total of 11 slices were taken from the center of the intervertebral disc, five slices above and below the center with an interval of 1 mm, constituting a total of 2,200 images for the development set and 990 images for the test set. The dotted red line shows the center of disc space and the orange dot the above and below the center of disc space with an interval of 1 mm

The CT scans of 204 consecutive patients who underwent both lumbar spine CT and abdominal CT within a 6-month interval between January 2014 and July 2021 were retrospectively retrieved from the databases of our hospital. After excluding 88 patients with acute burst fractures, infectious spondylitis, metastasis, or masses, 116 patients were included. Of these, seven patients for whom CT scan data were not recognized by the program were excluded. The final dataset used to develop the models included both lumbar spine and abdominal CT scans of 109 patients. Of these, data of nine patients who underwent CT scans after August 2020 were used as the temporal test set. The data of the remaining 100 patients were randomly split into 81 datasets for training and 19 for validation. In the development set of 100 patients, each patient had only one disc level and was randomly assigned to L1-L2, L2-L3, L3-L4, L4-L5, or L5-S1. The development set consisted of a total of 200 levels, including 100 levels for lumbar CT and 100 levels for abdominal CT. In the test set of 9 patients, all disc levels of the lumbar spine for each patient were analyzed. The test set consisted of a total of 90 levels, including 45 levels for lumbar CT and 45 levels for abdominal CT. For each level, a total of 11 slices were obtained from the center of the intervertebral disc, five slices above and below the center, with an interval of 1 mm (Fig. 1 and 2). A total of 2,200 images for the development set and 990 images for the test set were used.

**Fig. 3:**
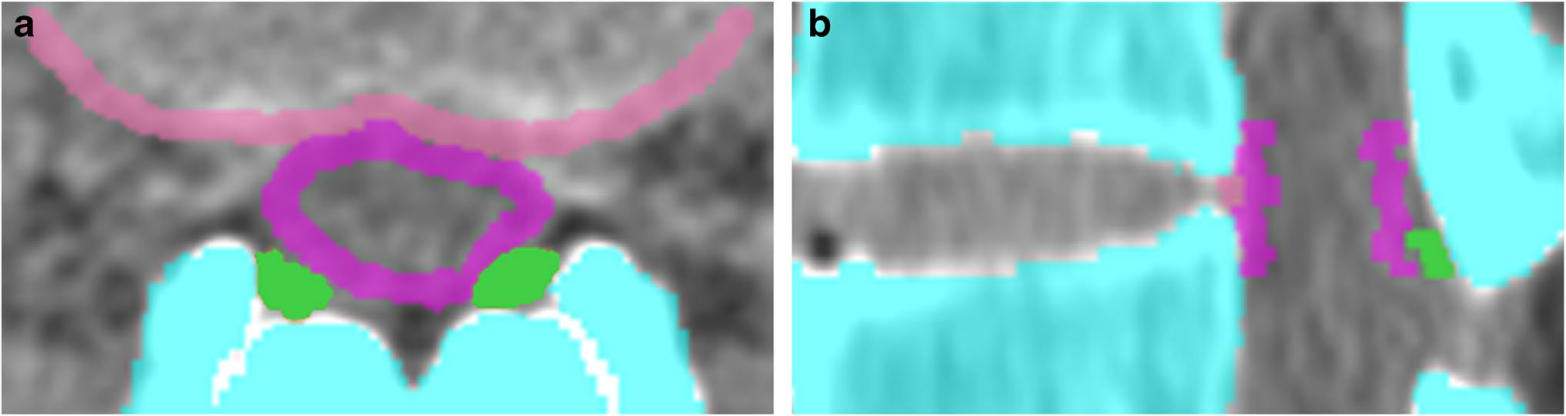
Representative images of the labeling program. The program automatically detects the bone of the lumbar spine (indicated by blue color in the image). Labeling of the dural sac area (pink), disc posterior margin (light pink), and ligamentum flavum (green) on an axial CT scan by the radiologists and students (**a**). Automatic reformatting of sagittal (**b**) image by the program

All scans were labeled by three radiologists (J.W.L. with 19 years of experience in musculoskeletal radiology, E.L. with 10 years of experience in musculoskeletal radiology, and B.R.K. with 4 years of experience in musculoskeletal radiology) with the help of two students. The radiologists and students delineated the DSA, disc posterior margin, and ligamentum flavum on each section of the 3,190 axial CT images (Fig. 3). A supervising radiologist reviewed each section and corrected the student-generated delineations.

### CT protocol

The most frequently performed CT scans were selected for this study, which were non-contrast-enhanced lumbar spine CT and contrast-enhanced abdominal CT. In this study, the CT data were collected over a long period and various CT scanners were used. Both non–contrast-enhanced lumbar spine CT and contrast-enhanced abdominal CT were performed using six CT scanners (Brilliance 64, Philips Healthcare, The Netherlands; IQon Spectral CT, Philips Healthcare, The Netherlands; iCT 256, Philips Healthcare, The Netherlands; SOMATOM Definition Edge, Siemens Healthineers; SOMATOM Force, Siemens Healthineers; SOMATOM X.cite, Siemens Healthineers). The scanning parameters of non–contrast-enhanced lumbar CT were a slice thickness of 1 mm with an interval of 0.5 mm, tube voltage of 120–150 kV, tube current of 200–450 mAs, pitch factor of 0.80–1.00, and rotation time of 0.4–1.0 s. Scanning parameters of contrast-enhanced abdominal CT were a slice thickness of 2 mm with an interval of 1 mm, tube voltage of 100–120 kV, tube current of 150–380 mAs, pitch factor of 0.60–0.99, and rotation time of 0.5 s.

### Data pre-processing

First, the centroids of each vertebral body were identified using the vertebra localization algorithm developed by Coreline Soft. Next, a curve passing through the estimated center was generated. It was assumed that the curve is a virtual spinal curve. Finally, image reformatting was performed for a total of 11 slices by selecting five upper and lower slices at 1-mm intervals based on the center of the intervertebral disc. Image reformatting means reconstructing an image in a plane direction perpendicular to the virtual spinal curve. When reformatting was performed, the thickness of the image was fixed at 1 mm.

### Model architecture

**Fig. 4:**
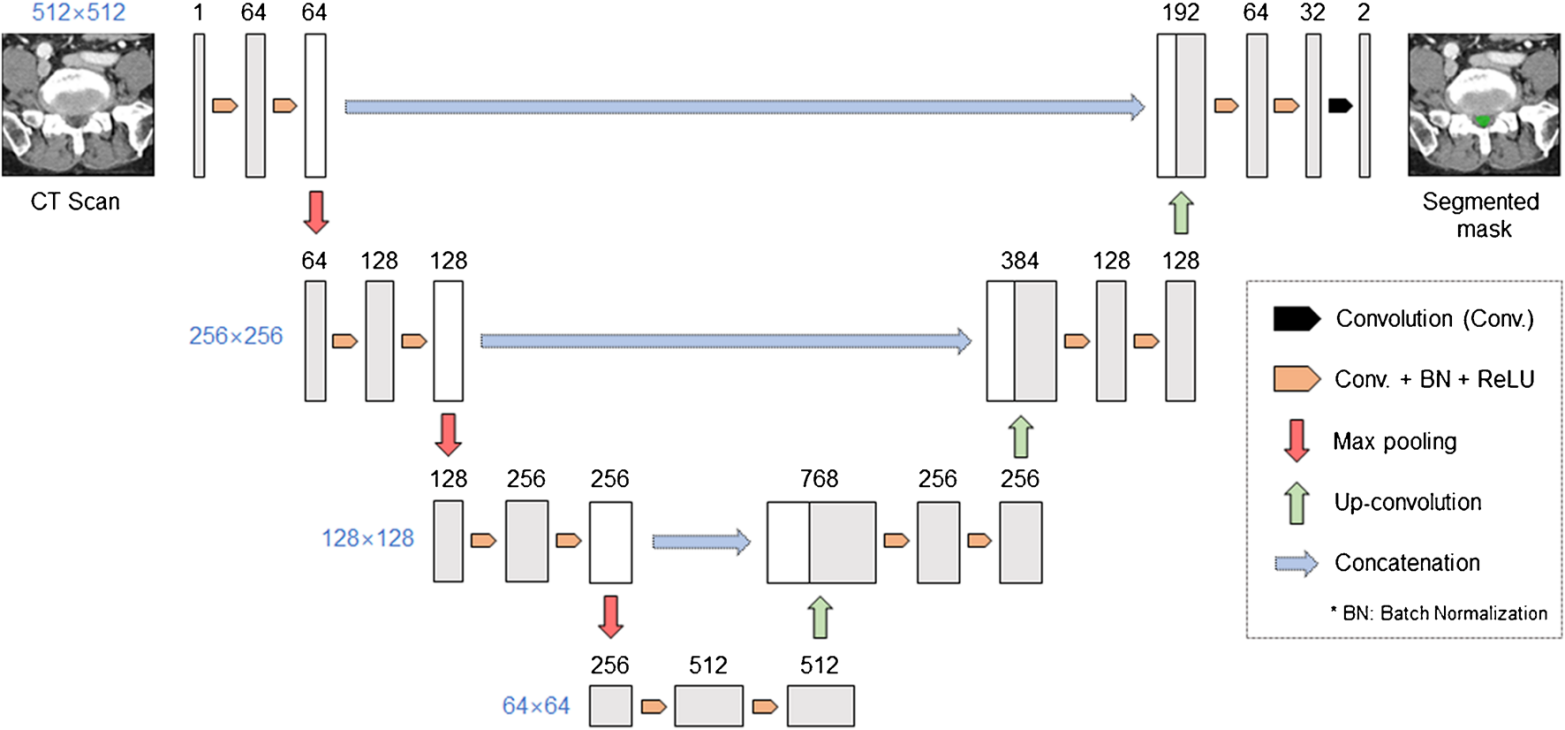
Overview of the proposed network architecture. The U-Net architecture was used as the model architecture. Each box corresponds to a multi-channel capability map. Convolution is a layer that applies a convolutional filter to the image and feature map to compress the information efficiently. Batch normalization is a technique that helps different features to have a consistent distribution, and ReLU is an activation function that maps the outputs of the layer to values greater than or equal to zero so that errors are evenly transmitted during the learning process. Max pooling is a method of generating a smaller feature map by taking the maximum value in each region of the feature map, and Up-convolution is a layer that increases the size by applying a convolution filter to the feature map. Concatenation stacks feature maps of the same size for a channel dimension

The U-Net architecture was used as the model architecture [19]. It extracts features of various image levels through a contracting path composed of convolution and pooling layers and through an expansive path composed of up-sampling layers and convolution layers. Subsequently, it performs detailed localization from the feature maps through an expansive path composed of up-sampling and convolution layers. In this process, by concatenating the feature maps derived from the contracting path to the feature maps of the expansive path, the segmentation performance is improved by using the features of various scales (Fig. 4).

### Model training

In this study, semi-supervised learning was applied to minimize labeling and maximize performance [20]. To apply semi-supervised learning, unlabeled data are required in addition to labeled data. The training dataset was constructed in the following way.

Each patient has five disc levels from L1-L2 to L5-S1. We labeled only one level and collected images for the other levels. Consequently, it was possible to obtain an unlabeled dataset that was approximately four to five times larger than the labeled data.

The model architecture required modification to apply semi-supervised learning. In general, U-Net has one encoder and one decoder. However, in this study, two decoders were connected to an encoder. The reason for using two decoders is to effectively use unlabeled data. Both decoders have almost similar architecture and differ only in the way they grow the size of the feature maps. When training on labeled data, they are trained independently. However, when training on unlabeled data, there is no ground truth; hence, the outputs of the two decoders serve to provide pseudo-labels for each other. In the inference phase, only one decoder is used. This process was implemented through a specially designed loss function and cross-entropy, which are generally used for segmentation model training, and the consistency loss that compares the outputs of the two decoders was used together. The L2 loss was used for consistency loss. For effective learning, we used the Adam optimizer. The epoch and learning rate were 50 and 0.001, respectively.

### Data augmentation

The dural sac, which is the segmentation target of the model, can have various shapes depending on whether stenosis is present. Therefore, elastic transformation has been used to apply spatial transformation to study various types of dural sacs. In addition, random scaling, rotation, Gaussian blurring, and Gaussian noise, which are commonly used techniques, were applied together to achieve a robust performance against various changes, such as image quality and protocol.

### Clinical study

For the clinical study, 200 levels from 20 patients who underwent both lumbar spine CT and abdominal CT within a 6-month interval between January 2020 and July 2021 were used. One musculoskeletal radiologist who had not participated in the previous labeling on program development was asked to delineate the narrowest DSA at each level of the lumbar spine. All slices at each level were measured using deep learning and were compared with the one that the radiologist considered the narrowest.

### Statistical analysis

The datasets were divided into two groups: normal (dural sac area ≥ 100 mm^2^) and stenosis (< 100 mm^2^). Model performance was evaluated by measuring the Dice similarity coefficient (DSC), intraclass correlation coefficient, sensitivity, specificity, and accuracy. DSC is one of the most used metrics for evaluating image segmentation in medical imaging [21]. Intraclass correlation coefficient was adopted to assess agreement in measurements for the following comparisons: deep learning algorithm versus the radiologist and abdominal CT versus lumbar CT. Manually segmented images were used as the ground truth for performance evaluation. Statistical analyses were performed using R Statistical Software (version 3.6.2, R Foundation for Statistical Computing, Vienna, Austria).

## Results

### Patient characteristics

We identified 109 patients who underwent both lumbar spine and abdominal CTs. The development dataset comprised 2,200 individual images from 100 patients. The validation dataset comprised 990 individual images from nine patients. The training and validation sets included 44 men and 56 women, and the test set included six men and three women. The mean age was 68 ± 13 years for the training and validation sets and 77 ± 7 years for the test set. The patient demographics and clinical characteristics of the training and validation sets are summarized in Table 1.

**Table 1:** Patient characteristics and distribution of development and temporal test sets

Category	Development Set*	Temporal Test Set	Total
Number of Patients	100	9	109
Age (y)†	68 ± 13	77 ± 7	69 ± 13
Sex			
Men	44 (88.0)	6 (12.0)	50
Women	56 (94.9)	3 (5.1)	59
Numbar of CT‡	200	18	218
Number of Levels§	200 (69.0)	90 (31.0)	290
Normal (DSA** ≥ **100mm^2^)	110 (75.3)	36 (24.7)	146
Stenosis (DSA < 100mm^2^)	90 (62.5)	54 (37.5)	144

Except where indicated, data are the numbers of patients, with percentages in parentheses

^*^The development set refers collectively to the training and validation sets

^†^Data are presented as mean ± standard deviation

^‡^Data are the number of CTs, including lumbar and abdominal CTs, for each patient, thus doubling the number of patients

^§^Data are the numbers of disc levels, including one level for the development set, thus equal to the number of CTs, and five levels for the test set, thus five times the number of CTs

### Dural sac segmentation performance of the deep learning algorithm

Table 2 presents a performance summary of the dural sac segmentation model for this dataset. The DSC of the deep learning algorithm was 0.85 ± 0.10, which indicates high segmentation performance. The segmentations generated by the deep learning algorithm overlapped significantly with the corresponding ground truth. Both abdominal and lumbar CT showed comparable performance with a DSC of 0.85 ± 0.09 and 0.85 ± 0.11, respectively. The intraclass correlation coefficient of the overall, abdominal, and lumbar CTs was 0.82 (95% confidence interval [CI]: 0.80, 0.85), 0.82 (95% CI: 0.79, 0.85), and 0.82 (95% CI: 0.77, 0.86), respectively. The measurement agreements between the radiologist and the deep learning algorithm were very good. In addition, the intraclass correlation coefficient between abdominal and lumbar CTs on deep learning was 0.89 (95% CI: 0.87, 0.91). The agreement between abdominal CT and lumbar CT was also good.

**Table 2:** Segmentation performance of deep learning algorithm in dural sac cross-sectional area of the lumbar spine

	DSC	ICC
Overall CT	0.85 ± 0.10	0.82 (0.80–0.85)
Abdomen CT	0.85 ± 0.09	0.82 (0.79–0.85)
Lumbar CT	0.85 ± 0.11	0.82 (0.77–0.86)

The numbers in parentheses are 95% confidence intervals, and data for DSC are shown as mean ± standard deviation. DSC, Dice similarity coefficient; ICC, intraclass correlation coefficient

### Performance of deep learning model for the classification of central canal stenosis on the lumbar spine

Table 3 presents the performance of the deep learning algorithm in diagnosing central canal stenosis of the lumbar spine. The proposed deep learning algorithm for diagnosing stenosis of the lumbar spine with dichotomous classification (normal or stenosis) showed overall accuracy of 84%, sensitivity of 72%, and specificity of 92%. In the dataset analysis, the algorithm showed accuracy of 85% and 83%, sensitivity of 78% and 66%, and specificity of 90% and 94% in abdominal and lumbar CTs, respectively. The accuracy and sensitivity of abdominal CT was better than that of lumbar CT.

**Table 3:** Performance of deep learning algorithm in diagnosing central canal stenosis of the lumbar spine

	Accuracy	Sensitivity	Specificity	Accuracy*
Overall CT	83.9 (81.5–86.2)	71.9 [281/391] (67.1–76.3)	91.8 [550/599] (89.3–93.9)	77.8 (67.8–85.9)
Abdomen CT	85.3 (81.8–88.3)	77.8 [147/189] (71.2–83.5)	89.9 [275/306] (85.9–93.0)	82.2 (68.0–92.0)
Lumbar CT	82.6 (79.0–85.9)	66.3 [134/202] (59.4–72.8)	93.9 [275/293] (90.5–96.3)	73.3 (58.1–85.4)
Severe stenosis CT†	85.4 (83.0–87.5)	57.6 [132/229] (51.0–64.1)	93.7 [713/761] (91.7–95.3)	77.8 (67.8–85.9)

Numbers in parentheses are 95% confidence intervals (CIs), and numbers in brackets are raw data. Accuracy, sensitivity, and specificity are presented as percentages

^*^Accuracy at the narrowest dural sac cross-sectional area (DSA) at each disc level. The smallest value of DSA among the 11 images of each level is considered to represent the stenosis of the level, constituting a total of 90 levels, and is divided into normal and stenosis based on 100 mm^2^

^†^ Performance of deep learning algorithm in diagnosing severe central canal stenosis of the lumbar spine; the data were divided into two groups: normal (DSA ≥ 75 mm^2^) and severe stenosis (DSA < 75 mm^2^)

As a further study of the severity of spinal canal stenosis, the data were divided into two groups: normal (DSA ≥ 75 mm^2^) and severe stenosis (DSA < 75 mm^2^) [3, 5]. The deep learning algorithm for diagnosing severe stenosis of the lumbar spine showed an accuracy of 85%, sensitivity of 58%, and specificity of 94% (Table 3).

### Performance comparison of deep learning model with the radiologist in the clinical study

Segmentation performance between the deep learning algorithm and a radiologist was compared in this clinical study because radiologists diagnose stenosis on CT images by selecting the narrowest slice at each level of the lumbar spine in real-world scenarios. In the clinical study, the DSC of the deep learning algorithm in the narrowest slice considered by the radiologist was 0.82 ± 0.01. The segmentations generated by the deep learning algorithm overlapped significantly with those of the radiologist at the same slice of each level. Both abdominal and lumbar CTs showed comparable performance, with a DSC of 0.82 ± 0.01 and 0.82 ± 0.01, respectively. When reviewing each CT image, the radiologist and deep learning detected the narrowest DSA to a similar extent among 200 slices (Table 4). In some cases (44.0%), deep learning was even better at identifying the narrowest slice than the radiologist. Therefore, deep learning can detect the narrowest DSA comparable to the findings of a radiologist.

**Table 4:** Comparison between deep learning and the radiologist in clinical study

	Number of slice*
Deep learning = Radiologist†	23 (11.5%)
Deep learning > Radiologist‡	88 (44.0%)
Deep learning < Radiologist§	89 (44.5%)
Total	200

^*^Number of the narrowest slice at each level between the radiologist and deep learning

^†^When the radiologist and deep learning select the same narrowest slice

^‡^When deep learning is better than radiologist for selecting the narrowest slice

^§^When the radiologist is better than deep learning in selecting the narrowest slice

### Representative image

**Fig. 5:**
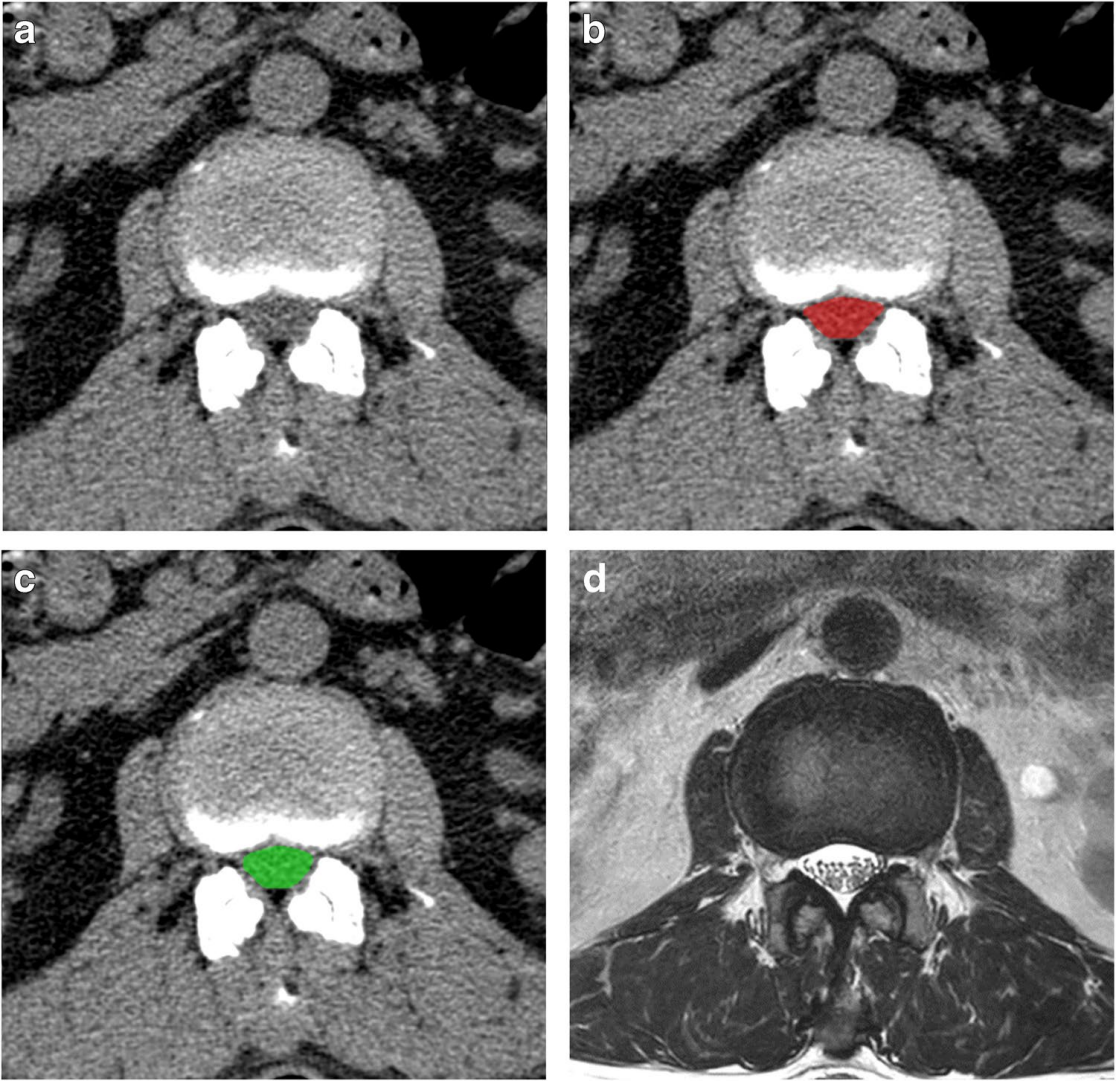
Representative cases of dural sac segmentation on a normal case of lumbar CT without contrast. The images of axial CT imaging (**a**) selected from the dataset are shown alongside their resulting segmentation of the dural sac by a manual rater (**b**, red) and the proposed algorithm (**c**, green). The dural sac area is 127.6 mm^2^ (**b**) and 124.2 mm^2^ (**c**), respectively. Figure 5d is an MR axial image of this case, which was taken within 6 months of the CT scan

**Fig. 6:**
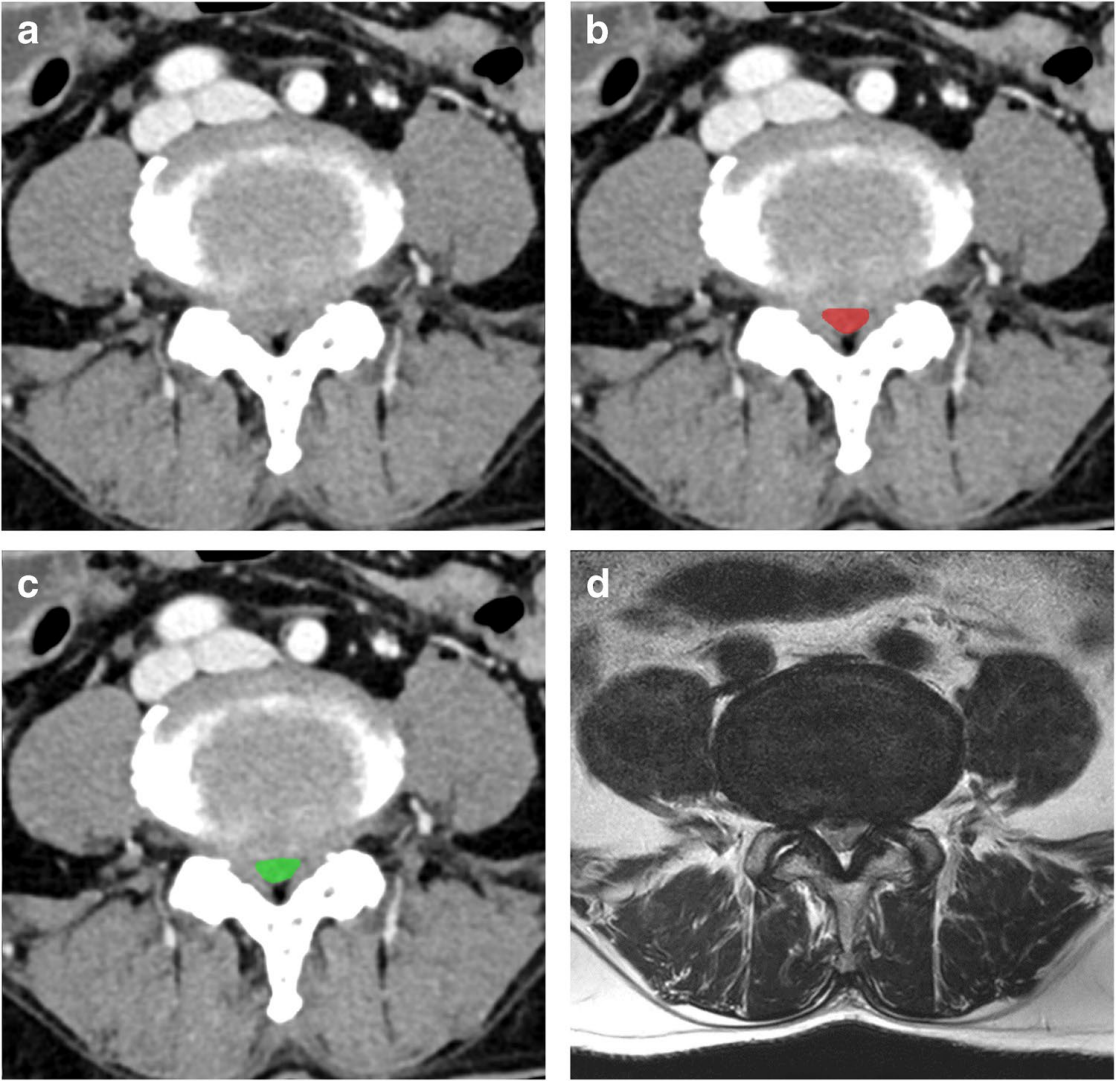
Representative cases of dural sac segmentation on a stenosis case with contrast on abdominal CT. The images of axial enhanced abdominal CT imaging (**a**) selected from the dataset are shown alongside their resulting segmentation of the dural sac by a manual rater (**b**, red) and the proposed algorithm (**c**, green). The dural sac area is 50.6 mm^2^ (**b**) and 45.6 mm^2^ (**c**), respectively. Figure 6d is an MR axial image of this case, which was taken within 6 months of the CT scan

Figure 5 is the normal case of lumbar CT without contrast, and Fig. 6 is the stenosis case using contrast on abdominal CT. As shown in Figs. 5 and 6, the algorithm identified the boundaries of the dural sac very well. This result indicates that there is a feature in the image that distinguishes the border of the dural sac from the surrounding structures, such as the disc posterior margin, bony canal, and ligamentum flavum. In Fig. 6, soft tissues, including the disc and ligamentum flavum, are enhanced to better delineate the dural sac area.

In addition, in Figs. 5d and 6d, we can see that the normal and stenosis cases on MRI appear similarly on CT; these images are taken from a subset of patients in our dataset (26 out of 109 patients who had L-spine MRI within a 6-month interval).

## Discussion

In this study, we developed a deep learning model to diagnose central canal stenosis of the lumbar spine using both abdominal and lumbar CTs. Our proposed algorithm could simultaneously detect all levels of the lumbar spine and evaluate the presence of central canal stenosis on both lumbar and abdominal CT images. Furthermore, our deep learning program could automatically delineate the DSA and diagnose lumbar canal stenosis with human-level performance.

While comparing the performance of our deep learning algorithm in diagnosing central canal stenosis between lumbar CT and abdominal CT, the accuracy of abdominal CT images was not inferior to that of lumbar CT. This is probably due to the presence or absence of contrast enhancement on CT. Lumbar spinal stenosis is caused by a decrease in the dural sac area, the space for the spinal cord and nerve roots, as a result of changes in the bone and soft tissue elements surrounding the spine [22–24]. Therefore, how accurately the bony structures and soft tissue elements are detected is important in defining the boundaries of the dural sac area. In this study, we selected the most frequently performed CT scans, which were non-contrast-enhanced lumbar spine CT and contrast-enhanced abdominal CT. There are no meaningful differences in bone structure between abdominal CT and lumbar CT, but soft tissue structure changes depending on the presence or absence of contrast agent. Contrast agents in abdominal CT alter soft tissue attenuation to better differentiate the dural sac from the surrounding structures [25]. Therefore, it allows for better detection of extradural compressing lesions, such as disc herniation or ligamentum flavum hypertrophy. Detecting surrounding soft tissue structures enhanced by contrast ultimately makes a difference. Furthermore, abdominal CT has twice as thick slices as lumbar CT with a lower kilovoltage, which implies less image noise and better visualization of soft tissue boundaries.

Some studies have concluded that CT and MRI show very close agreement in the assessment of lumbar spinal stenosis [26–31]. One study showed 96.6% agreement between MRI and contrast CT in the diagnosis of spinal stenosis [31]. In a meta-analysis [30], sensitivity of CT and MRI for diagnosing lumbar central canal stenosis was comparable ranged from 0.81 to 0.97 for MRI, from 0.70 to 1.0 for CT. In addition, one study comparing the effectiveness of CT and MRI in delineating the soft tissues of the lumbar spinal stenosis suggested that CT is better at detecting the osseous structure of spinal canal and excellent delineation of the ligamentum flavum compared with MR [32]. Although MRI remains the imaging modality of lumbar stenosis and CT has limitations in the evaluation of the spinal canal because CT cannot see the nerve roots of the central canal, this suggests that it can be a good screening tool if central canal stenosis is detected on CT before MR imaging for patients who are not clinically suspected. In addition, some older patients with symptomatic lumbar spinal stenosis have a neuro-stimulator or pacemaker, which precludes them from undergoing MRI. Our deep learning algorithm can help these patients.

Abdominal CT is a frequently performed imaging test for a variety of clinical indications, and unsurprisingly, abdominal CTs are performed on patients who have central canal stenosis of lumbar spine without typical symptom, especially older patients. Symptoms of lumbar spinal stenosis may be ambiguous in older patients, and many of them are found to have radiologic lumbar spinal stenosis but are asymptomatic or manifest with misleading pain without evidence of progressive neurogenic claudication, the hallmark clinical symptom of lumbar spinal stenosis [33, 34]. However, central canal stenosis can become serious if left untreated. Prompt treatment is required to slow the progression of stenosis even in the early stages. If central canal stenosis is left untreated for a long period of time, it may worsen and cause serious symptoms, such as low back pain and weakness in the extremities. However, despite its clinical importance, many spinal abnormalities, including stenosis, are often missed on abdominal CT.

With recent advances in CT technology, the post-processed thin section sagittal reformation has allowed the assessment of the lumbar central canal stenosis on abdominal CT. One study suggested that abdominal CT studies can detect lumbar spine abnormalities with an accuracy of 91.7/99.8% on a per patient/per abdominal finding basis [35]. Nevertheless, lumbar spine abnormalities on abdominal CT are often not reviewed routinely. Another study [36] found that < 10% of fractures are mentioned in the radiology report, and another study [35] reported that abdominal CT reports mentioned only 14.1% of the abnormal findings in the lumbar spine. Although sagittal reformations can be easily examined, the interpreting radiologists often review axial images. This may be due to a lack of awareness of the clinical significance of identifying spine abnormalities and belief that incidental and unexpected findings may not be considered relevant or important. Therefore, our deep learning program can help detect lumbar spinal stenosis on abdominal CT by automatically calculating the DSA of the lumbar spine and diagnosing the central canal stenosis. Further, it will help radiologists reduce their workload and save the time when reporting abdominal CT. With our program, patients who have central canal stenosis but undergo abdominal CT for asymptomatic or misleading pain can receive an explanation for their symptoms and may avoid additional costly evaluation. In addition, our algorithm also showed good accuracy in cases of high-grade stenosis, thus suggesting an additional benefit that it may also identify patients with clinically significant severe stenosis.

Our deep learning program showed low sensitivity, which means that the algorithm tends to measure DSA smaller than the radiologist. However, because of the high specificity of our deep learning program, if this program diagnoses lumbar spinal stenosis on abdominal CT, further evaluations, such as additional clinical history taking or MRI, can be strongly suggested. If stenosis is determined with our deep learning program, it may be meaningful to consider whether central canal stenosis is the cause of pain.

This study had some limitations. First, we had relatively small datasets because patients who underwent both abdominal and lumbar CTs within a 6-month interval were selected. Second, we did not evaluate the deep learning algorithm using completely independent external datasets. Model training with additional data from other institutions is required to improve the generalizability of the algorithm. Third, the reference standard used in this study was CT images segmented by experts and not MRI. As MRI is still the current imaging modality in patients with low back pain, it is necessary to know how much agreement exists between the two modalities. However, the number of patients who underwent abdominal CT and lumbar spine MRI with a short interval was small in our hospital. Between January 2014 and July 2021, only 26 of 109 patients underwent L-spine MRI within a 6-month interval. Patients usually do not undergo abdominal CT and lumbar MRI within a short period of time. Therefore, further studies including large-scale multi-center study are warranted to evaluate lumbar stenosis using MRI as an additional reference standard. Prospective research is also needed to determine the clinical utility and therapeutic impact of our algorithm.

In conclusion, our deep learning algorithm automatically assessed lumbar spinal stenosis on both lumbar and abdominal CT images. The diagnostic performance of abdominal CT for lumbar spinal stenosis is comparable to that of lumbar CT.

## Data Availability

The data that support the findings of this study are available for scientific purposes from the corresponding author upon reasonable request.
